# Late Intragastric Erosion of a Laparoscopic Adjustable Gastric Band With Associated Abscess Formation: A Case Report

**DOI:** 10.7759/cureus.106083

**Published:** 2026-03-29

**Authors:** Ulrica Armbrister, Jasndeep Kaler, Shashawna S Drum Christie, Tanisha Jain, Tissha Soogrim, Frederick Tiesenga

**Affiliations:** 1 College of Medicine, Windsor University School of Medicine, Cayon, KNA; 2 College of Medicine, St. George's University, St. George, GRD; 3 General Surgery, West Suburban Medical Center, Chicago, USA

**Keywords:** band removal, bariatric surgery, gastric band erosion, laparoscopic adjustable gastric banding, late postoperative complications, obesity

## Abstract

One of the most prevalent public health issues seen in the United States is obesity. Bariatric surgery, particularly innovations such as laparoscopic adjustable gastric banding (LAGB), is a manageable and reversible technique employed to attain weight loss for patients who are severely obese.

LAGB, like any medical procedure, is associated with serious but rare complications. Some of the short-term complications include pleural empyema, gastric perforation, and diaphragmatic injury. Conversely, some long-term complications include, but are not limited to, gastric band erosion, band slippage, migration of the gastric band into the gastric lumen, and gastric band adhesions.

This paper probes into the management of a patient who was found to have gastric band adhesions through the lens of a comprehensive case involving a 52-year-old male presenting with this specific complication nine years post-LAGB. Challenges linked with the diagnosis and management of gastric band adhesions will be methodically highlighted. The patient presented with extensive comorbidities, including his BMI, which instigated the necessity for a multidisciplinary approach regarding treatment and postoperative care. This case allows for a comprehensive level of understanding as it pertains to the complications of LAGB and further accentuates the need for a thorough care strategy in patients who undergo bariatric surgery. The findings in this research concentrate on the need for follow-ups and attentiveness during the postoperative period in an effort to not only improve patient outcomes but also to limit the instances of such complications.

## Introduction

Obesity remains a major global health concern and is associated with increased risks of cardiovascular disease, diabetes mellitus, and premature mortality. In the United States, projections indicate that nearly half of adults will be classified as obese by 2030 [1]. Bariatric surgery has become the most effective and durable treatment for severe obesity, producing sustained weight reduction and improvement in obesity-related comorbidities [2].

Among the bariatric procedures developed, laparoscopic adjustable gastric banding (LAGB) gained popularity due to its minimally invasive approach, adjustability, and reversibility [3]. The procedure involves the placement of an inflatable silicone band around the proximal stomach to create a small gastric pouch, thereby restricting food intake and promoting early satiety. Despite its initial appeal, long-term follow-up studies have revealed considerable complications and failure rates, prompting a global decline in its use [4].

Complications of LAGB can be broadly categorized as early (e.g., gastric perforation, infection, or hemorrhage) or late (e.g., band slippage, pouch dilation, port malfunction, and band erosion). Band erosion, also known as intragastric migration, is a relatively uncommon but serious late complication, occurring in approximately 1-3% of patients [4,5]. The pathogenesis is multifactorial, involving chronic ischemia, mechanical stress, and inflammatory changes at the band-stomach interface [4]. Clinical manifestations are often nonspecific, including abdominal pain, dyspepsia, nausea, or localized infection, and diagnosis typically requires endoscopic or radiologic confirmation [5]. Management of band erosion generally necessitates surgical removal of the device to prevent further morbidity such as abscess formation, peritonitis, or sepsis. However, delayed presentations years after the initial surgery can complicate both diagnosis and operative management [5].

This report presents the case of a 52-year-old male who presented nearly a decade after LAGB placement with gastric band erosion and associated abscess formation. The case underscores the diagnostic challenges, multidisciplinary management, and postoperative considerations involved in addressing late LAGB complications.

## Case presentation

A 52-year-old African American male with a past medical history of morbid obesity, type 2 diabetes mellitus, hypertension, hyperlipidemia, and heart failure presented to a suburban hospital with complaints of left groin pain. His past surgical history was significant for laparoscopic gastric band placement in 2016 at a community hospital and a left foot ulcer debridement in 2020. He denied any history of tobacco, alcohol, or illicit drug use.

At the suburban hospital, diagnostic imaging performed for evaluation of his symptoms incidentally revealed findings concerning gastric band erosion with an adjacent abscess. Upon discussion of the diagnostic results, the patient further endorsed experiencing intermittent abdominal discomfort for which he had not previously sought medical evaluation. The attending physician at the suburban hospital contacted the surgical team at a neighboring community hospital regarding the concerning findings, and the patient was prepared for transfer. He was started on intravenous cefepime and vancomycin prior to transfer for further surgical management. The specific imaging studies and reports from the suburban hospital were not available for review at the time of presentation.

Upon arrival at the community hospital, the patient reported persistent mild abdominal discomfort along with left groin pain, which had prompted his initial presentation. He stated that he had experienced intermittent abdominal discomfort since approximately 2017 but had not sought medical attention, as the pain was initially mild and self-limited. However, these symptoms gradually progressed and began causing significant discomfort approximately two weeks prior to presentation. He also described a boil in the left inguinal fold that had been present for several days, noting that it was mobile, warm, and tender to the touch. He rated his pain as five out of 10 and endorsed decreased appetite, intermittent fevers, chills, and mild nausea. He denied vomiting, changes in bowel habits, chest pain, or shortness of breath.

On arrival at the community hospital, vital signs were as follows: blood pressure 160/108 mm Hg, heart rate 114 beats per minute, temperature 99.6°F, respiratory rate 18 breaths per minute, and oxygen saturation 90%. His weight was 395 lb (179.2 kg), and his height was 6′2″ (1.88 m). Laboratory studies are summarized in Table 1. On examination, the patient was alert and awake, with mild discomfort. His abdomen was soft and mildly tender to palpation, particularly in the right flank region, with diffuse warmth. Inspection of the left inguinal fold revealed localized erythema, induration, and fluctuance consistent with cellulitis and possible abscess formation. The remainder of the physical examination was unremarkable.

**Table 1:** Laboratory results at presentation

	Patient’s Values	Reference Range
White Blood Cell (k/mm^3^)	12.4	4.0-11.0
Platelets (k/mm^3^)	238	150-450
Red Blood Cell (m/mm^3^)	4.98	4.34-5.60
Hemoglobin (g/dL)	13.8	13.0-17.0
Hematocrit (%)	41.8	38.6-49.2
Mean Corpuscular Volume (fL)	84.1	80.0-100.0
Mean Corpuscular Hemoglobin (pg)	27.7	26.0-34.0
Mean Corpuscular Hemoglobin Concentration (%)	33.0	32.5-35.8
Red Cell Distribution Width (%)	17.3	11.9-15.9
Mean Platelet Volume (fL)	8.2	6.8-10.2
Neutrophils (%)	81.0	40.0-60.0
Lymphocytes (%)	9.2	20.0-40.0
Monocytes (%)	8.7	2.0-8.0
Eosinophils (%)	0.8	1.0-4.0
Basophils (%)	0.3	0.5-1.0
Neutrophils Absolute (k/mm^3^)	10.1	1.7-7.7
Lymphocytes Absolute (k/mm^3^)	1.1	0.6-3.4
Monocytes Absolute (k/mm^3^)	1.1	0.3-1.0
Eosinophils Absolute (k/mm^3^)	0.1	0.0-0.5
Basophils Absolute (k/mm^3^)	0.0	0.0-0.2

**Figure 1:**
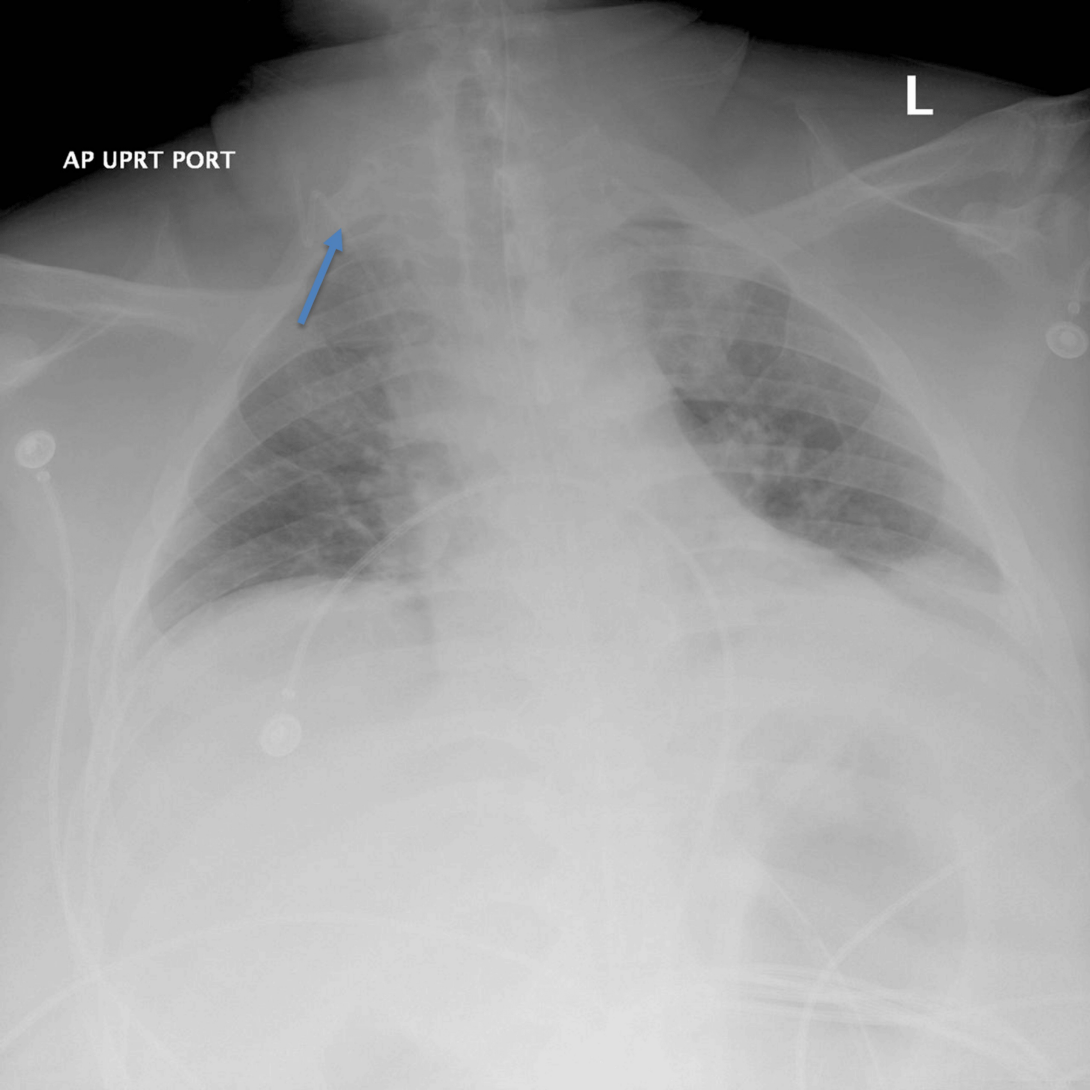
AP chest X-ray demonstrating a properly positioned right internal jugular central venous catheter The blue arrow indicates the position of the right internal jugular central venous catheter. The portable AP chest X-ray confirms the course of the CVC inserted via the right neck. The radiopaque catheter tracks inferiorly and medially, with its tip appropriately positioned within the SVC, projected just above the level of the right atrium. AP, anteroposterior; CVC, central venous catheter; SVC, superior vena cava

Given the concern for gastric band erosion and infection, the patient was evaluated by the surgical team. After discussing the risks, benefits, and alternatives, he consented to proceed with operative intervention. Intraoperatively, a laparoscopic approach was initially attempted for lap-band removal but was converted to an open procedure due to poor visualization. The gastric band was not visible with the use of ultrasound, prompting intraoperative esophagogastroduodenoscopy (EGD), which demonstrated near-complete erosion of the band into the gastric lumen. The eroded band, which was bile-stained and adherent to the gastric wall, was removed using a transgastric approach, and the resulting gastrotomy was repaired in two layers with Graham patch reinforcement. Two Jackson-Pratt drains were placed, and a Penrose drain was draped under the midline laparotomy to allow for additional drainage. A nasogastric (NG) tube was inserted for gastric decompression. Additionally, a right internal jugular central venous catheter line (Figure 1) was inserted, and the decision was made to keep the patient nil per os (NPO) until a follow-up upper gastrointestinal (UGI) study could be performed five days postoperatively. Incision and drainage of the left groin were also performed, though no purulent material was identified.

The patient tolerated the procedure moderately well but experienced mild intraoperative hemodynamic instability, requiring placement of a right internal jugular central line and transfusion of one unit of packed red blood cells. He was subsequently transferred to the intensive care unit for postoperative monitoring, where his blood pressure stabilized. Postoperative vital signs were as follows: blood pressure 129/75 mm Hg, heart rate 91 beats per minute, temperature 98.1°F, respiratory rate 18 breaths per minute, and oxygen saturation 97%. Infectious disease was consulted for management, and intra-abdominal and device cultures were obtained. While awaiting culture results, the patient was initiated on broad-spectrum antimicrobial therapy with Zosyn (piperacillin-tazobactam) and Diflucan (fluconazole). Antibiotic coverage was to be adjusted according to final culture and sensitivity results.

**Figure 2:**
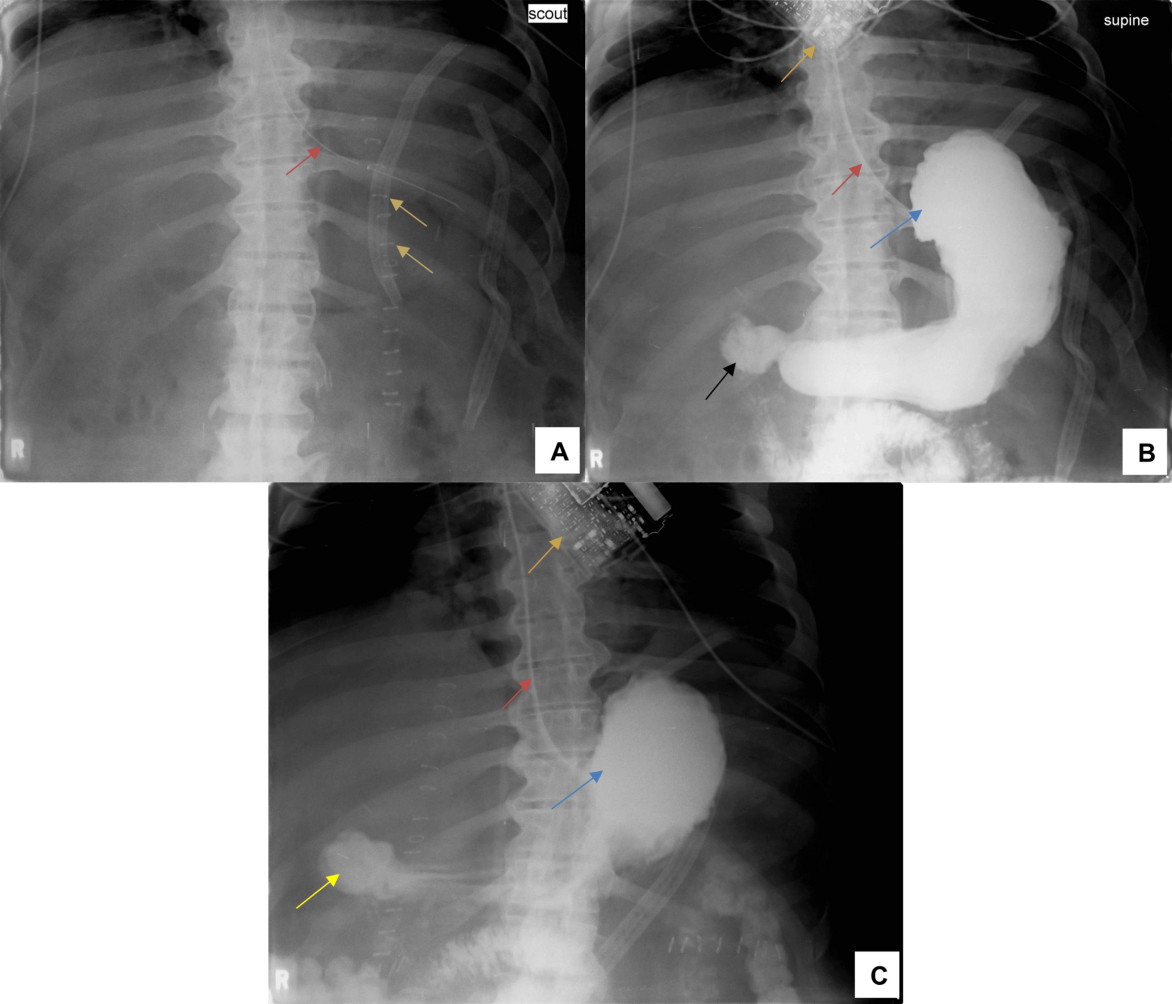
Supine UGI radiograph demonstrating postoperative anatomy following laparoscopic gastric band removal A: Scout abdominal radiograph prior to contrast administration. A radiopaque enteric NG tube is visualized coursing along the esophagus toward the stomach (red arrow). Metal clips can be seen near the gastroesophageal junction (yellow arrows). No pneumoperitoneum or abnormal bowel gas patterns are identified. B: Upper GI contrast study AP view following administration of Gastrografin. The contrast-filled gastric lumen is clearly visualized (blue arrow), with smooth filling of the gastric body and antrum. Contrast passes normally into the duodenal bulb (black arrow). The orange arrow shows the lap band device (no longer in its normal position). No extraluminal contrast is seen, and the absence of leak is noted. Position of the NG tube (red arrow). C: The stomach remains uniformly opacified (blue arrow), with unobstructed flow into the duodenal sweep. The non-opacified excluded gastric remnant persists in its expected location (yellow arrow), consistent with intact anatomy and no fistulous communication. No extraluminal contrast is identified. The red arrow indicates the position of the NG tube. The orange arrow indicates the lap band device, no longer connected. UGI, upper gastrointestinal; NG, nasogastric; GI, gastrointestinal; AP, anteroposterior

A follow-up UGI study conducted five days after surgery demonstrated no evidence of leak (Figure 2). Based on these findings, the surgical team elected to remove the NG tube, and a clear liquid diet was initiated and well tolerated.

At one week postoperatively, final culture results from intraoperative specimens revealed scant growth of Candida albicans and heavy growth of carbapenem-resistant Enterobacteriaceae (CRE). Based on these findings, the Infectious Disease team adjusted the antibiotic regimen from Zosyn (piperacillin-tazobactam) to Avycaz (ceftazidime-avibactam) for appropriate coverage, while continuing Diflucan (fluconazole) as previously prescribed. The patient remained hemodynamically stable, and his postoperative course continued to improve. Plans were made for an interval EGD in six to eight weeks to evaluate for gastric healing.

## Discussion

This case illustrates the unexpected shift of clinical focus from a localized soft-tissue infection to a more complex surgical consideration involving a previously placed gastric band. The patient initially presented to the emergency department with complaints of a painful, erythematous groin abscess associated with swelling and tenderness. Additionally, he reported several days of intermittent abdominal pain, decreased appetite, fever, chills, and nausea. Given these symptoms, abdominal imaging was obtained, which revealed erosion of a previously placed LAGB with evidence of localized infection. In response to these findings, the patient underwent laparoscopic gastric band removal.

LAGB is a minimally invasive bariatric surgical procedure designed to induce weight loss by restricting food intake. During the procedure, an adjustable silicone band is placed around the upper portion of the stomach, creating a small proximal gastric pouch that promotes early satiety and limits caloric intake [6]. Although generally considered safe, long-term complications such as band slippage, erosion, infection, or port malfunction may occur, sometimes necessitating surgical removal, as observed in this case.

This case demonstrates a delayed presentation of a late gastric band complication nearly a decade after placement. The patient’s chronic, nonspecific gastrointestinal symptoms, such as abdominal discomfort, nausea, and reduced appetite, had been disregarded until incidentally uncovered during diagnostic workup for the groin abscess. Such findings emphasize the importance of maintaining a high index of suspicion in individuals with a history of bariatric surgery who present with even subtle gastrointestinal complaints.

LAGB was once considered an attractive bariatric option due to its minimally invasive approach, adjustability, and reversibility. However, long-term studies have revealed significant complication and reoperation rates. Up to 50% of patients may eventually require revisional surgery, with approximately 25% to 40% undergoing complete band removal due to erosion, slippage, or intolerance [6,7]. Common late complications include band slippage (4-13%), pouch dilation (5-10%), erosion (1-2%), esophageal dilation (up to 10%), and port- or tubing-related problems (2-6%) [8]. The pathogenesis of these complications is multifactorial, involving mechanical stress, over-tightening of the band, chronic inflammation, local ischemia, and nonadherence to dietary recommendations.

The pathophysiology of band erosion, as seen in this patient, involves chronic mechanical pressure on the gastric wall, leading to ischemia, inflammation, and eventual transmural necrosis [9]. Although gastric band erosion is relatively uncommon, it is among the most serious complications associated with LAGB. Clinical manifestations often include dull epigastric pain, nausea, vomiting, dysphagia, or, in cases with port involvement, localized infection [8]. Diagnosis typically relies on imaging studies such as UGI series or computed tomography, with endoscopy providing definitive confirmation of mucosal erosion [10].

Band erosion incidence varies across studies but typically ranges from 1% to 3% of patients. A systematic review of 25 studies, including 15,775 patients, reported an overall erosion incidence of 1.46% [4]. Another study showed a gastric band erosion rate of approximately 3.4% [10]. Band-related infections are less frequently reported; however, the U.S. Food and Drug Administration reported that 4% of LAP-BANDs were removed in their post-approval review due to infection [11].

Management of band erosion invariably requires device removal. Surgery remains the gold standard, as conservative therapy rarely provides lasting benefit and may risk worsening infection or perforation. In this case, laparoscopic removal was appropriately undertaken, resulting in symptom resolution and prevention of further sequelae. However, literature indicates that 40% to 60% of patients experience significant weight regain within two years following band removal if no revisional bariatric procedure is pursued [6,10]. Postoperative care should therefore include multidisciplinary follow-up encompassing nutritional counseling, behavioral therapy, and consideration of alternative bariatric strategies such as sleeve gastrectomy or Roux-en-Y gastric bypass.

The declining prevalence of LAGB reflects recognition of its limitations. Data from the International Federation for the Surgery of Obesity and Metabolic Disorders (IFSO) demonstrate that LAGB accounted for approximately 42% of bariatric procedures in 2008 but decreased to <5% by 2018, primarily due to high complication and reoperation rates [12]. This global trend underscores the shift toward more effective and durable metabolic procedures with improved long-term safety profiles.

This case reinforces the importance of comprehensive evaluation in patients with a history of bariatric surgery, particularly when presenting with vague or seemingly unrelated abdominal complaints. Early recognition and prompt surgical management of LAGB complications can prevent severe outcomes such as sepsis or gastric perforation. As bariatric surgery continues to evolve, clinicians should remain aware of the long-term risks associated with adjustable gastric banding and ensure appropriate patient education, follow-up, and counseling on revisional options.

## Conclusions

LAGB is associated with significant long-term complications and reoperation rates, including rare but serious events such as intragastric erosion and abscess formation. This case highlights a delayed presentation nearly a decade after band placement, underscoring the importance of maintaining clinical vigilance in patients with a history of bariatric surgery who present with nonspecific gastrointestinal symptoms. Early recognition and prompt surgical intervention are critical to preventing severe morbidity. Long-term surveillance, thorough symptom evaluation, and multidisciplinary follow-up are essential components of post-bariatric care, particularly for patients with legacy gastric bands. As the use of LAGB continues to decline, awareness of its late complications remains important for timely diagnosis and optimal management.
